# Social Determinants of Cognitive Health and Cognitive Health Care Planning

**DOI:** 10.1093/geroni/igaf122.1175

**Published:** 2025-12-31

**Authors:** Alexa Allan, Abigail Stephan, Elizabeth Muñoz

## Abstract

Social determinants are critical to shaping our path toward health equity. In fact, research has highlighted healthcare, education access, social and community context, economic stability, and neighborhood and built environment as key factors in health and well-being across the lifespan. This symposium highlights contextual and healthcare-based determinants to advance our understanding of their role in shaping cognitive health, emphasizing midlife and older adulthood. This symposium will include presentations using data from the Healthy Aging in Neighborhoods of Diversity Across the Life Span Sleep Study (HANDLSleep) and a project through the South Carolina Alzheimer’s Disease Research Center (SC-ADRC) that examines Black/African American families’ intergenerational discussions around Alzheimer’s disease and related dementias (ADRD). These studies explored the psychosocial and contextual factors of sleep and cognition in a sample of Black and White middle-aged and older adults in Baltimore, Maryland, and the psychological and social factors influencing conversations and decision-making in cognitive healthcare across generations of Black/African American adults in South Carolina. The objectives of the proposed symposium are the following: (1) discuss the associations observed between neighborhood, leisure, and cognition using ambulatory data, and (2) discuss the role of social support and healthcare planning in cognitive health. Allan and colleagues explored the association between neighborhood quality and cognitive function using ambulatory measures. Sardina and colleagues examined the coupling of leisure activities and cognition. Simon and colleagues explored whether researcher-facilitated family conversations increased ADRD knowledge. Lastly, Stephan and colleagues explored family members’ perspectives on cognitive healthcare by rurality.

